# Distributed Data Service for Data Management in Internet of Things Middleware

**DOI:** 10.3390/s17050977

**Published:** 2017-04-27

**Authors:** Ruben Cruz Huacarpuma, Rafael Timoteo de Sousa Junior, Maristela Terto de Holanda, Robson de Oliveira Albuquerque, Luis Javier García Villalba, Tai-Hoon Kim

**Affiliations:** 1 Cybersecurity INCT Unit 6, Decision Technologies Laboratory—LATITUDE, Electrical Engineering Department (ENE), Technology College, University of Brasília (UnB), Brasília-DF, CEP 70910-900, Brazil; rubencruzh@unb.br (R.C.H.); desousa@unb.br (R.T.d.S.J.); robson@redes.unb.br (R.d.O.A.); 2 Department of Computer Science, University of Brasília (UnB), Brasília-DF, CEP 70910-900, Brazil; mholanda@unb.br; 3 Group of Analysis, Security and Systems (GASS), Department of Software Engineering and Artificial Intelligence (DISIA), Faculty of Computer Science and Engineering, Office 431, Universidad Complutense de Madrid (UCM), Calle Profesor José García Santesmases, 9, Ciudad Universitaria, Madrid 28040, Spain; 4 Department of Convergence Security, Sungshin Women’s University, 249-1 Dongseon-Dong 3-ga, Seoul 136-742, Korea; taihoonn@daum.net

**Keywords:** Internet of Things (IoT), IoT middleware, data collection, data aggregation, real time, NoSQL

## Abstract

The development of the Internet of Things (IoT) is closely related to a considerable increase in the number and variety of devices connected to the Internet. Sensors have become a regular component of our environment, as well as smart phones and other devices that continuously collect data about our lives even without our intervention. With such connected devices, a broad range of applications has been developed and deployed, including those dealing with massive volumes of data. In this paper, we introduce a Distributed Data Service (DDS) to collect and process data for IoT environments. One central goal of this DDS is to enable multiple and distinct IoT middleware systems to share common data services from a loosely-coupled provider. In this context, we propose a new specification of functionalities for a DDS and the conception of the corresponding techniques for collecting, filtering and storing data conveniently and efficiently in this environment. Another contribution is a data aggregation component that is proposed to support efficient real-time data querying. To validate its data collecting and querying functionalities and performance, the proposed DDS is evaluated in two case studies regarding a simulated smart home system, the first case devoted to evaluating data collection and aggregation when the DDS is interacting with the UIoT middleware, and the second aimed at comparing the DDS data collection with this same functionality implemented within the Kaa middleware.

## 1. Introduction

In the near future, the Internet of Things (IoT) is expected to connect an ever increasing number of smart and embedded devices, giving way to new opportunities to build applications that are more integrated to real-time situations in the physical world. It is estimated that the number of interconnected devices will reach 50 billion by 2020 [1,2,3], considering that there are currently over four billion mobile phone users who already have the ability to access the Internet. Intelligent systems and devices, such as smart phones, smart transport means (for instance, cars, bikes, boats), smart factories, smart machines and even smart clothes (for instance, glasses and watches), are already connecting and interacting automatically with sensors that generate and transmit data without human intervention, as well as with actuators able to intervene in the physical world.

While IoT offers new opportunities in application development, the management of large volumes of data produced in this environment continues to be a challenge. For example, it is worth considering a city transport monitoring system, with several sensors for the lights, cameras and poles, and where every 5 s, each sensor sends data posts of 160 bytes in length. In a single day the volume of data produced by each of these sensors would be 2.64 MB, in a month, 81.74 MB and, in a year, 962.40 MB. Thus, if this monitoring system comprises around 10 thousand sensors, it can generate more than 9.6 TB per day. In this class of application, the number of sensors tends to grow every day.

A common architectural component supporting these simultaneous and parallel IoT applications, which we define as IoT instances, is the IoT middleware, a system that effectively manages the various heterogeneous components that must interconnect and interoperate [4,5]. Specifically, the IoT middleware is a software layer between the physical layer of devices and their interfaces and the application layer that integrates the logic and control of the IoT instance. In order to facilitate the integration and the communication of heterogeneous components, it is required of the IoT middleware to provide a set of programming abstractions, including device discovery, access and administration services, among others. For this class of systems, large-scale data transmission throughout the Internet is a standard requirement, given the need of sharing data pervasively in the network. Managing the corresponding enormous volume of data is still a formidable challenge. In addition, each middleware can use a specific data format, such as, for example, the XML format in some middleware and the JSON format in others. Consequently, it is challenging to integrate all data coming from different IoT environments, with different formats, arising in large volume and high variety, and this challenge requires better approaches on how to collect, process and store these data; otherwise, it remains for every application to cope with the related issues.

For managing the large volume of data within IoT environments, IoT middleware systems implement particular data collectors, using particular data formats and different storage architectures, ranging from centralized relational database management systems to NoSQL distributed systems. In general, these data collectors are logically tied and strongly coupled to the middleware architecture, as in, for example, EcoDif [6], Xively [7], OpenIoT [8], RestThing [9], WSO2 [10] and Kaa [11]. In a common structure for an IoT system instance, one or more of these middleware will manage IoT devices that must send data to an Internet application server, this being the application server and the managed devices owned by the same organization. However, it is foreseen that future IoT systems will involve applications to access data collected from devices owned by different individuals and organizations. In such future systems, largely distributed and reliable data management services will be required to cope with heterogeneity and support data analytics and learning processes for IoT.

In this paper, to tackle the challenge of managing the data produced in IoT network instances, a different approach is taken in the form of a DDS to collect and process data coming from different IoT middleware. Thus, the central objective of our proposal is to provide IoT data management services specified and developed to work with different IoT middleware, i.e., not being coupled to a specific middleware. This service provider is then required to integrate different IoT middleware simultaneously to collect and manage IoT data. An expected result is that for new middleware development, rather than creating for each one a new component for data management, they can use the proposed data service, which would be ever improved for managing large volumes of data. Additionally, the proposed service may support Big Data produced by IoT instances and, as a result of processing this massive amount of generated data, can generate knowledge that is crucial to drive better decisions that are common to various IoT instances.

The proposed DDS is also designed to support multiple data sources simultaneously, since it is not coupled to a specific middleware. This allows DDS to serve different IoT middleware, simultaneously, to collect and manage IoT data, which is interesting since improvements in managing large volumes of data within the proposed data service benefits all of the client middleware instead of each middleware having to create a new component. Furthermore, the proposed service may provide support to acquiring knowledge from Big Data produced by IoT instances, a feature that seems to be crucial in making common decisions regarding various IoT instances [12]. Regarding Big Data support, the proposed DDS is designed to collect huge data volumes from distributed sources and then to store large datasets in different physical nodes, for the sake of scalability. Furthermore, by means of its data aggregation component, DDS can deal with historical data. To cope with the variety of data formats used by IoT platforms, including data objects, which are not relational, in the proposed DDS communications interface, incoming data is converted to a generic structure (JSON message). Moreover, since many IoT objects generate data at high rate data streams, specific data flow processing can be instantiated within DDS to cope with this velocity aspect of IoT Big Data.

Thus, the proposed data service is developed to distribute processing and to adapt at different levels to computing parallelism, according to the needs of IoT instances. The DDS comprises two main components: data collection and data aggregation, both of them able to collect and process data in distributed and parallel processing. While the collection component should have the ability to efficiently capture data produced from a large network of devices, the data aggregation component is required to efficiently summarize huge volumes of data in real-time. These requirements imply that the data service must tackle several challenges, such as storing data, avoiding processing bottlenecks, dealing with device/sensor heterogeneity and presenting high throughput, as described in this paper. To deal with a huge volume of data, the DDS architecture is characterized as a distributed architecture to process and store data. Data heterogeneity in this architecture is handled both by a communications interface that converts different data formats and a metadata creation module, able to manage different metadata characteristics.

To validate the proposed architecture and its supporting protocols, a corresponding prototype was developed and implemented. Then, this prototype was submitted to testing and simulations by means of which we verified its functional characteristics and its performance. The proposed service evaluation is firstly carried out within the simulation of a smart home system that is used as a scenario for analyzing data collection and aggregation functionalities in the interactions of DDS with the existing UIoT middleware [13]. Then, the same scenario serves to compare the performance of DDS data collection compared with a similar data collector within the Kaa middleware [11].

The remainder of this paper is organized as follows: Section 2 discusses requirements regarding Big Data management within IoT; Section 3 is a brief survey of related works; in Section 4, the distributed data service is proposed for supporting the management of data in IoT, the components supporting this proposed service are outlined together with their functional specification; Section 5 presents the implementation of this DDS with the choice of software modules and the processing topology; Section 6 presents a case study (smart home system) to validate our approach; Section 7 presents the observed results, which are discussed in Section 8; and finally, Section 9 presents the conclusions and possible future works.

## 2. Big Data Management for IoT Middleware

The ever-increasing number of IoT instances generates a continuous flow of large amounts of data chunks that are usually time-stamped and possibly geo-stamped. These data can come in the form of simple key-value pairs, or they may contain rich audio/image/video content. The structural complexity of data chunks and the data flow characteristics can considerably vary according to data content. This massive and heterogeneous dataset, generated by IoT environments, presents a number challenges regarding storage and processing. In this context, the data volume generated and consumed by IoT applications can be characterized as Big Data and the IoT instances as a Big Data sources [14,15].

As IoT devices/sensors capture real-time events, also possibly involving mobility, these devices/sensors produce a high volume of highly varied data at high speeds. Subsequently, this collected data needs to be analyzed in order to extract knowledge. Even when this processing can be performed in a traditional computing environment (centralized servers), this operation mode is prone to capacity limitations regarding processing power and storage volume [16,17]. In counterpoint, there are emerging distributed environments with more adequate features, including scalability means and parallel processing, to overcome the cited limitations of centralized architectures.

Most definitions of Big Data have been focusing on the so-called 3V characteristics [18]: volume, variety and velocity. However, the concept of Big Data has evolved to comprise the so-called 7V characteristics [19], thus including veracity or validity, value, volatility and visualization. Veracity or validity requires that data are verifiable and truthful. Value is related to the importance, worth or usefulness of information derived from exploiting Big Data. Volatility refers to data meaning, which is constantly changing, and visualization relates to all means that make a vast amount of data comprehensible in a manner that is easy to understand and read. Moreover, there are several other V’s that may be considered: viability, volatility, vitality, and others. IoT data characteristics are compatible with these multiple V definitions as argued by some authors [20,21,22,23], and the IoT middleware is required to bring answers to the corresponding challenges, by way of its modular structure and functions, as described in the following subsections.

### 2.1. IoT Middleware

**Figure 1:**
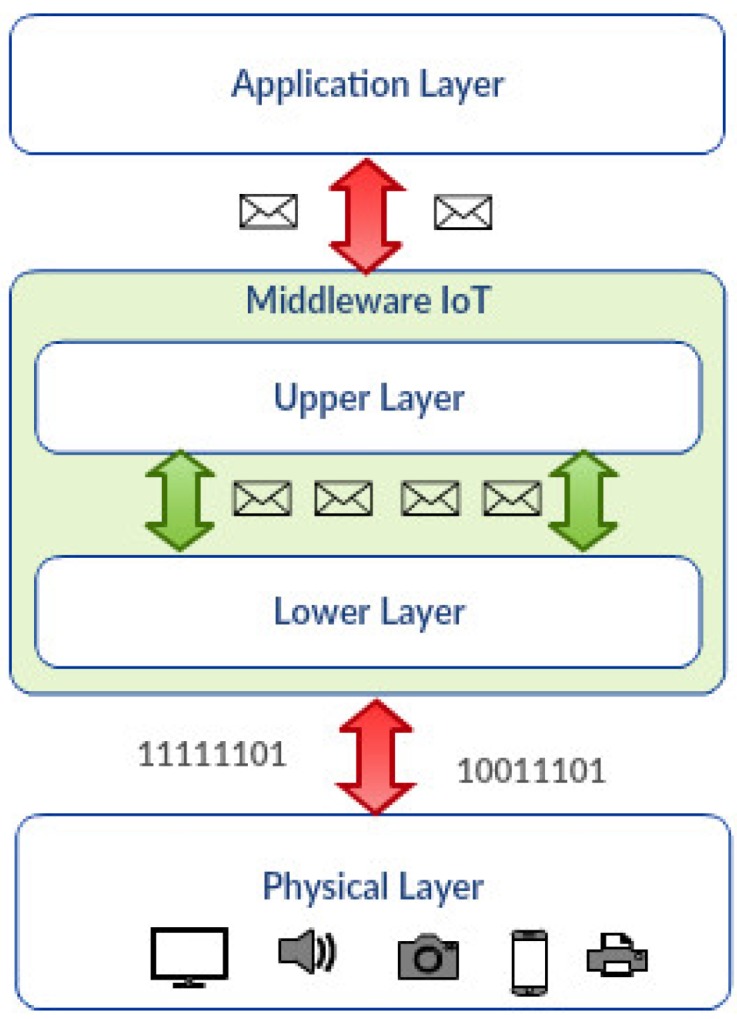
IoT architecture.

The IoT middleware is a system piece that effectively manages the various heterogeneous components (sensors, aggregators, actuators and others) that comprise an IoT environment [4,5]. Figure 1 shows a general view of the different layers that compose an IoT middleware. In general, the upper layer is a component that has direct interaction with the application layer within the IoT architecture. The application layer receives request messages and sends responses related to services provided by the middleware. The lower layer interacts with the physical layer and exchanges binary information and control commands/responses with the physical devices.

In order to meet requirements related to IoT, an IoT middleware provides for applications the necessary abstractions of things and offers multiple services based on these abstractions. These abstractions and related services are used for addressing interoperability and adaptation across heterogeneous devices, context awareness, scalability, device discovery, management of large volumes of data and the security aspects of the IoT environment [24]. Among the middleware features, one of paramount importance is the ability to hide details of different technologies, thus helping programmers to focus on the development of the control logic for IoT applications. Specifically, the IoT middleware helps to bring together a multitude of devices and data in a way that enables developers to create and deploy new IoT services without having to write different codes for each kind of device or data format [25]. For this reason, IoT middleware packages are gaining importance in recent years due to their major role in simplifying the development of new services and the integration of legacy technologies into new ones [26], exempting the programmer from the need to have proficiency in the rich set of technologies adopted by the lower layers.

According to [27], a common IoT middleware functional component is a module devoted to managing the data volume, which is expected to be very large indeed. Therefore, it is really important to create novel methods to find, fetch and transfer data in this context, solving challenges that involve querying, indexing, process and transaction handling models, considering that they must deal with identification, positional, environmental, historical and descriptive data, as presented in [28]. In general, the necessary component uses database management systems (DBMS) to manage data, as well as specific software technologies to process the huge volume of data.

### 2.2. Database Requirements for IoT Environments

The Internet of Things presents a new set of challenges for database management systems, such as absorbing large volumes of data in real time or near real time, processing events as they flow rather than on a schedule of fixed intervals and dealing with significantly larger volumes of data than those often encountered in enterprise applications [17]. In order to properly handle IoT data and its requirements, a critical choice concerns the necessary database or set of databases.

There are many factors to keep in mind when choosing a database for IoT data management, and these factors do not always align with the characteristics of more traditional database systems [17]. In this context, among the many requirements to consider, some of the most important are scalability, ability to absorb data at high rates, schema flexibility, integration with analytics tools and costs.

Ideally, IoT databases would be linearly scalable, so, for example, adding one more server to a 10-node cluster would increase throughput by 10%, but coordination and communication operations usually preclude this linear extension. However, considering that IoT is a distributed system, IoT databases will usually be distributed ones unless the application collects only a small amount of data that will not grow substantially. Distributed databases can run on commodity hardware and scale by adding new servers instead of swapping out a server for a larger one.

An IoT database should also be fault tolerant and highly available. If a node in the database cluster is down, the database service should still be able to accept read and write requests. Distributed databases make copies, or replicas, of data and write them to multiple servers. In the case of the failure of one of the servers that stores a particular dataset, then one of the other servers storing a replica of this dataset can respond to a read query. Regarding write requests, if the server that would normally accept a request is down, another node in the server cluster can accept the request and forward it to the target server when it is back to an operational status.

Moreover, since IoT databases must be as flexible as required by IoT applications, it is noticeable that NoSQL databases, either key-value, columnar and document databases, easily accommodate different data types and structures without the need for predefined, fixed schemas. NoSQL databases are a valuable option when it is necessary to accommodate multiple data types and those data types will likely change over time.

In relational database systems, which are mainly storage-centric, the data volume generally is collected from predefined and finite sources and stored according to strict normalization rules for relationships. In this kind of database, the transaction management mechanisms apply the so-called ACID—Atomicity, Consistency, Isolation, Durability - properties in order to enforce overall data integrity [29,30]. NoSQL databases try to solve some of the traditional database limitations by relaxing some of the ACID properties, given that relaxing certain constraints may be a good measure for several applications. For example, NoSQL databases can perform quite well in circumstances where consistency constraints are not necessary [31]. By doing so, these NoSQL databases can become tolerant to network partitions, thus enabling adding servers to the setup when the number of devices/sensors increases, instead of adding more capacity to a single server [32].

In the context of IoT, columnar database features seem to be suitable for managing IoT data compared to other types of NoSQL databases. First, since key-value databases are totally schema-less, the storing of metadata definitions regarding IoT data can be difficult. Oppositely, columnar databases have column families to support metadata creation. Second, IoT data are not characterized to present many relationships (in the traditional sense); consequently, a graph database can be less suitable for IoT data, while a columnar database can, if needed, efficiently support a reasonable number of relationships. Finally, document and columnar databases can accommodate different data types and structures without the need for predefined, fixed schemas.

### 2.3. Data Processing Flow Requirements for IoT

In order to deal with the complex environment of IoT and its associated Big Data, it is essential to define appropriate techniques to support the structural complexity and performance requirements for processing data flows within this environment. This involves issues in the articulation of techniques, such as messaging systems and real-time computing. In the IoT context, messaging systems can be used to collect data coming from numerous devices/sensors and being processed by different subscribed clients, while real-time systems are very important to process real-time data coming from IoT instances.

Messaging in this context is defined as the exchange of messages among computing programs conveying specially-formatted data describing events, requests and replies. This is performed by means of a messaging server that provides a communications interface for client programs. This server also provides administrative and control functionalities, including data persistence and reliability [33]. There are two major messaging system models: the point-to-point and the publish-subscribe models [33]. In the point-to-point model, source programs send messages to queues, and the receivers, which are registered with some specific queues, can asynchronously retrieve the messages and then acknowledge them. The publish-subscribe model is based on the use of topics that can be subscribed to by several clients. Messages that are sent to topics are then received in an asynchronous way by all of the subscriber clients.

Real-time systems are defined as those systems in which the correctness of the system depends not only on the logical result of computation, but also on the time at which the results are produced [34]. There are three major components, as well as their interplay, which characterizes real-time systems: time, reliability and environment [35]. These components are crucial to avoid serious harm, both economical or in terms of loss of lives.

## 3. Related Works

Much effort has been made in the area of IoT data storage and processing, indicating that one central objective in this domain is to efficiently gather data generated by heterogeneous devices, then processing and storing both original and resulting data in a persistent data store. As mentioned before, to achieve this, IoT middleware systems usually implement a data collector.

In [36], a data collector, specifically projected for an IoT environment, is available through a REST API. Data are received in a message array format, and the collector splits these into single message packets and authenticates the sensor. After having been identified, the packets are put into a message queue in order to be processed. Before storing data in the database, a preprocessing task is carried out. Specific handling protocols exist to run on data coming from a given class of sensors, such as for verifying the relevance of data.

Web ecosystem of physical devices, EcoDiF [37] is a IoT middleware that has a publish-subscribe component that manages the registration of devices and performs the receiving of information coming from devices and sensors. Based on Java API specifications for RESTful web services (JAX-RS), it uses a RESTEasy implementation for service creation for receiving information. Data from the sensors are converted to the XML format and sent via HTTP PUT messages to be stored in a MySQL database.

The Xively platform [38] has a so-called ‘time series’ component that provides services for the collection, management and querying of data from devices. This component stores the data in a cloud architecture based on MySQL and Cassandra databases. While similar to EcoDif, Xively is based on REST principles and web standards. In Xively, data are organized into feeds, data points and data streams. A feed is the data of a monitored environment (i.e., a living room). Data streams represent data sent by a particular sensor within a feed (i.e., the temperature of the monitored environment), and a data point represents a single value of a data stream at a given time.

In [39], an Intelligent Transportation System (ITS), based on IoT technology, unfolds in three stages: data collection, data transmission and information processing. In the collection stage, Radio Frequency Identification Technology (RFID) is used to identify and recognize objects.

The OpenIoT middleware [8] supports a discovery and collection service for sensor data coming from mobile sensors. These services are achieved through a publish-subscribe middleware called Cloud-based Publish-Subscribe for IoT (CUPUS). This middleware uses a cloud database allowing parallel subscriptions. The data collection is done through serial ports, by means of UDP and HTTP requests.

The RestThing middleware [9] comprises a data collector devoted to embedded devices and a database that is responsible for storing the data and the characteristics differentiating the data sources. The storage is based in XML and JSON files.

The WSO2 (Web Services oxygenated) middleware [10] has a monitoring module named “business activity”. This module can collect data streams and process them in batch, real-time, interactive and predictive modes. Moreover, it is able to receive data from almost any event source through java agents, JavaScript clients, regarding IoT events. Furthermore, it publishes events through an API for real-time, batch or combined processing. Similar to other middleware, such as Xively, WSO2 uses a distributed storage database, such as Cassandra.

The Kaa project middleware [11] implements data collection through a specialized application to synchronously collect data for a specified IoT application. This application is developed according to the required endpoint. For this reason, the Kaa platform provides a set of options to specify the IoT data structure coming from endpoints. The collected data can be stored into a MongoDB or Cassandra database.

As a common data collection characteristic, the mentioned IoT middleware implement their own data collector component, although each one uses different storage systems ranging from the traditional centralized relational database to distributed NoSQL systems. It is important to highlight that these data collectors are tightly coupled to the respective IoT middleware architectures. Though these different IoT middleware systems have common features, especially in terms of integration and interoperability between heterogeneous physical devices, their feature implementations are designed for the specific middleware in which they operate.

The approach presented in this paper differs from the described approaches in that our DDS is designed to work with different IoT middleware, non-intrusively. Moreover, the proposed DDS is designed with parallel and distributed processing ability to support the large volume of data from IoT environments. Thus, the proposed DDS required functionalities and performance are studied to allow this DDS to perform both as an IoT data storage and a data manager for IoT data. This is supported by data collection and aggregation components that are intended to store and manage IoT data either in real-time and batch processing.

In our approach to the challenge of managing data produced from different IoT middleware, in order to cope with these heterogeneous middleware, the proposed DDS defines different components for data collection and aggregation. Inside each of these components, there is a specialized module to deal with metadata complexity and with the concurrent requirements of batch and real-time processing. In this sense, the proposed DDS architecture is similar to the Lambda architecture [40] for IoT data management, which comprises three layers, namely the batch, speed and serving layer. In DDS, the same functionality of Lambda’s batch and speed layers is integrated into a single streaming engine designed for handling both batch and real-time processing, and ingesting the related volumes of data, a feature that is possible due the temporal nature of IoT data. This integration overcomes the complexity of programing in two different distributed platforms, such as the speed and batch layer, to produce compatible batch and real-time outcomes.

## 4. Distributed Data Service for IoT Middleware

Since data generated by the sensors and devices of an IoT network must typically be delivered to storage and processing system servers, then we define a data management model for systems in which data come from a large number of devices and are funneled into the DDS for further processing. Thus, the DDS has the capacity to support, simultaneously, multiple connections with different IoT middleware; a feature of its communication interface.

**Figure 2:**
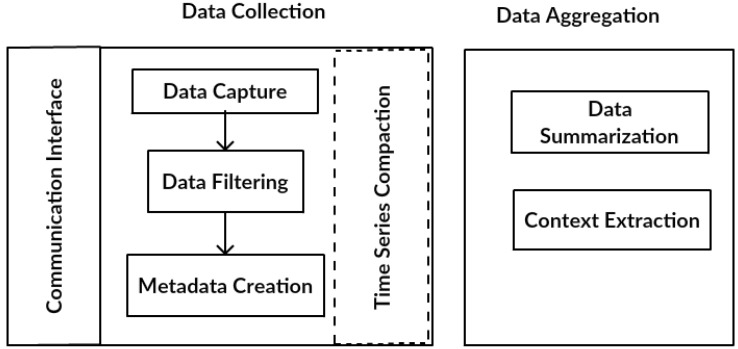
DDS components.

The DDS also comprises two main components (Figure 2): data collection and data aggregation. The data collection component receives data coming from different IoT middleware. In addition, the data collection stores captured data in the database to be used as input for the aggregation component. Stored historical data can be reprocessed in the future. While data are being collected, the data aggregation component summarizes the collected data in different periods.

The data collection component comprises five modules:

- Communication interface: responsible for establishing communications between each middleware and the DDS. This interface makes it possible to translate the messages used by a middleware to the message structure managed by DDS.
- Data capture: carries out the collection of messages exchanged throughout the IoT middleware layers.
- Data filtering: verifies the domain of the messages collected by the data capture module analyzing if the readings from the devices/sensors are among the values specified for them. The messages outside of the domain are discarded.
- Metadata creation: obtains important metadata such as timestamp, geo-location, unit of measurement and type of data. Other metadata can be included in the collection, according to the characteristics of IoT environments and applications.
- Time series compaction: this module groups the collected data, reorganizing it based on a time window. This module can be configured according to the volume and speed of data generation, in regards to the number of items collected by the data capture module.

The data aggregation component is responsible for the process in which the collected data are expressed in a summarized form. In general, the aggregated information is more significant than an isolated reading of a device. For example, the average number of readings for a device/sensor for a time period can be more significant than an isolated reading at a specific moment in time. A common guideline for data aggregation is to obtain more information about specific groups, with specific variables, as, for example, the time period or the function performed by each device/sensor. The data aggregation component comprises two modules:

- Data summarization: responsible for summarizing the data being collected. This component’s main objective is to reduce the data query volume. The aggregation of data reduces the number of lines to be retrieved, thereby improving the performance of consultations.
- Context extraction: responsible for the extraction of context and environment variables, such as, for example, the location of the devices/sensors, among others.

### 4.1. Data Collection Component

**Figure 3:**
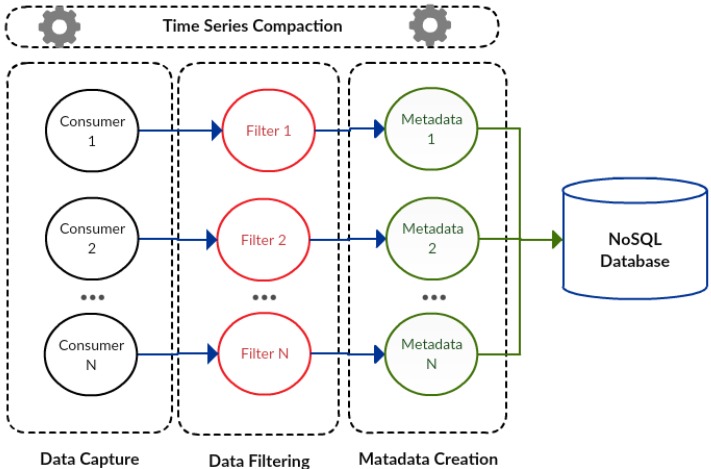
Data collection component.

The data collection component (Figure 3) is designed using a message system, a real-time computation system and a data storage system. The data capture module (consumers) of this component uses the message system, while the filter (filters) and metadata creation (metadata) modules use the real-time computation system. In addition, the series data compaction module was integrated within the data store system.

In Figure 3, processes related to consumers, filters and metadata are designed to handle large processing volumes since they can adapt to different parallel computing levels depending on environmental needs. These processes are executed according to a specific data flow, beginning within the consumer, passing through the filter to reach the metadata creator. The modules for data capture, data filtering and metadata creation can be instantiated in multiple different processes, each one with its respective consumers, filters and metadata.

The communication interface is responsible for establishing communications between an IoT middleware and the DDS. Since this interface must translate the messages used by each middleware to the message structure managed by DDS, it allows an IoT middleware to specify fields of messages sent to the DDS, so this service is enabled to transform these fields and send them through a specific channel for the desired purpose. Besides this, the data communication interface does not interfere with the normal flow of the IoT middleware data.

**Figure 4:**
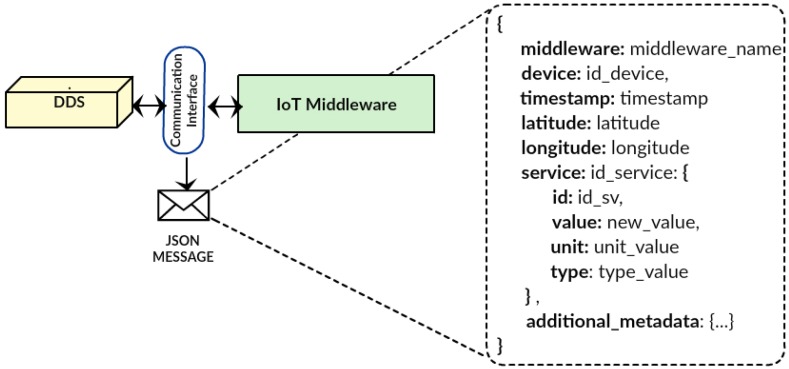
Communication interface between the DDS and the IoT middleware.

Figure 4 illustrates the DDS interaction with an IoT middleware through the communications interface module, for defining the communication port and JSON messages specifically for this IoT middleware. Then, each registered middleware must send a copy of every exchanged message, and the DDS listens to this port to capture the copies. Using JSON for this operation enables the reception of messages from different middleware, as the message structure containing the device/sensor in question is known. To cope with the heterogeneity of IoT data, most IoT middleware systems use JSON messages that differentiate several data sources. In DDS, the used JSON structure includes different attributes, such as: middleware name, device identifier and service identification, which includes the state variable and the new value of the reading. This JSON message also includes metadata, such as geo-location and type of data, and for more detailed metadata, an additional attribute is included in the form of a list of metadata describing specific data for particular IoT applications.

Data capture is designed to carry out the collection of messages exchanged within an IoT middleware. After being captured, the data are stored in the DDS without any modification, in order to keep a historical data log, whose retention time can be configured. This means that data coming from IoT environments is stored without any processing and can be removed according to the retention time. This data capture module performs several simultaneous processes using publish/subscribe communications and has the capacity to support different data sources (IoT middleware) publishing information in specific communication channels. Meanwhile, other processes can subscribe to the information that they want to receive from a communication channel. The basic interaction model uses an event service for signatures’ storage and management and efficient message delivery. This so-called broker represents a mediator between publishing and consumer processes. The broker sends messages to each consumer process, so that the publish processes do not need to know about consumer processes and vice versa. This constitutes a loosely coupled interface structure between IoT middleware systems and the DDS.

Data filtering verifies the domain of the messages collected by the data capture module, a feature that is realized through distributed processes executed in parallel. Data filtering requires querying the information database to make decisions according to filtering rules. For example, the verification analyzes if the readings from the devices/sensors are in the range of values specified for them. The messages that do not match the rule are discarded. The data filtering carried out according to a specific type of packaging by which the IoT middleware includes a plurality of devices/sensors. The filtering methods include the input data and a deploy rule, which allows implementing the rule by means of at least one process to be executed. These filtering processes can be executed in parallel, according to the demands of the IoT environment.

Metadata creation obtains relevant metadata from filtered messages, such as: the location in which the devices/sensors are operating, the time when a measurement was carried out and the specification of included data, which are frequently used to describe device/sensor measurements. Similar to the data filtering module, this module can be distributed and executed in parallel. In our proposal, we define two metadata groups: required and additional metadata. The required metadata are specified by time, geo-location, unit of measurement and type of data, which are considered essential in order to describe data coming from IoT environments. The required metadata fields are organized into a schema with predefined relationships to support efficient retrieval of data at later stages of processing. Typically, the measurement of each device/sensor must add metadata describing the data formats, a feature that is important for later query response and optimization, which implies that query processing has to adapt to a wide range of data formats that may not fully be known beforehand. Furthermore, metadata can be used to describe IoT data, IoT environments and IoT applications. For example, some metadata can be used to describe a specific domain application, such as an IoT application for agriculture. In order to receive additional metadata, an attribute named “additional metadata” is defined as a list of specific metadata to describe, for instance, a particular application domain.

The time series compaction module aims at grouping and sorting data within a certain time window, an important feature that can be configured according to the characteristics of the computational environment, improving its performance. Data compaction and reorganization aim to obtain significant gain in processing high volume device/sensor data and to save corresponding storage space. In this context, the compaction module groups can be configured according to the volume and speed of data generation, as a result of the items collected by the data capture module.

**Figure 5:**
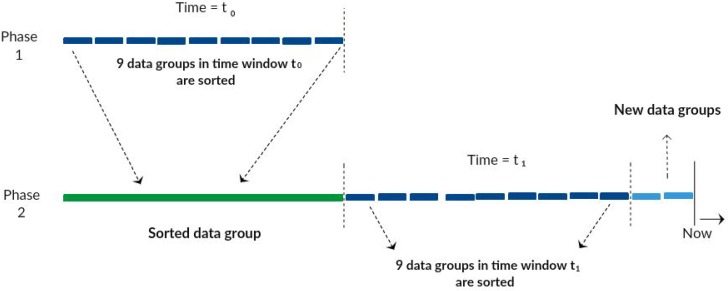
Example of time series organization.

Figure 5 gives an example of data compaction during a small time window, when there is a constant intense data flow, and also in a larger time window for less intense traffic. The idea is that, independent of the time window, the set of data will be grouped and then ordered continuously. In Phase 1, the time window t_0_ presents nine found data groups. In the second phase, the nine groups of data in the time window t_0_ are now sorted and compacted, while in time window t_1_, nine data groups are to be compacted, and two new data groups are arriving to be processed.

### 4.2. Data Aggregation Component

**Figure 6:**
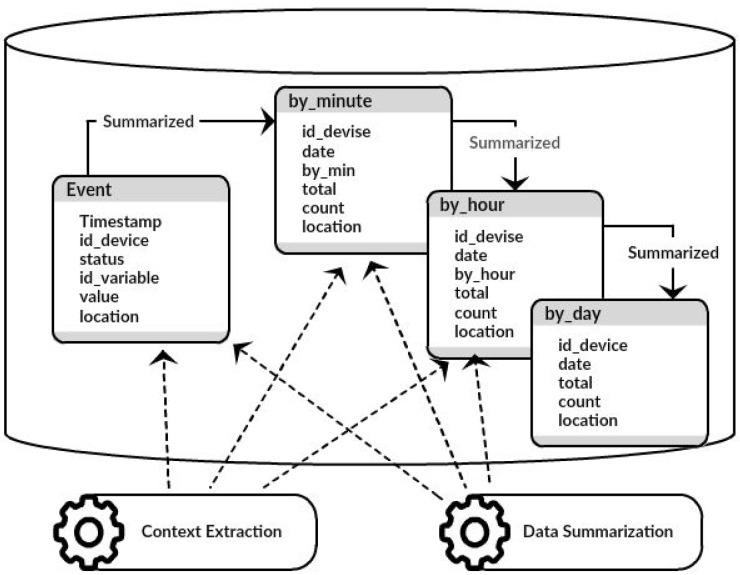
Data aggregation component.

While the data collection component receives data from the IoT middleware, the data aggregation component performs preprocessing of the collected data. The two processes run simultaneously, but each producing, accessing and verifying data at its own pace. These processes execute ceaseless queries for data in order to improve processing when dealing with huge volumes of data in real time. Nonetheless, the preprocessing module performs micro-querying over the database at intervals of minutes, hours and days, thus producing data summarization throughout these periods of time. As a result, this consists of another service for IoT applications, so these applications for processing a huge data volume in real-time are not required for querying over raw data, but rather over the data summarized by the aggregation component (Figure 6).

Data aggregation is one of the solutions to the problem of providing efficient solution processes to respond to real-time requests, considering resource limitations. The messages collected with the data collection component have metadata fields, including location, time and information about the data source. While the collection component is constantly receiving raw device/sensor data, these data are also being aggregated and summarized for individual sensor recordings. In the aggregation process, the data streaming from devices/sensors is separated for the modules’ data summarization and context extraction.

The data summarization module represents the grouping of a set of raw device/sensor data over a period of time. This reduces memory and computational cost related to massive amounts of real-time data produced by devices/sensors. For this reason, the semantic representation of the summarized data is as important as the annotation of the raw stream data, so this module reduces and creates this new form of representation. Since the data collection component stores data being collected into an event table, which is monitored by the data aggregation component, later the module can produce results that are stored into an aggregated table. Figure 6 shows the summarized tables by_minute, by_hour and by_day. The by_minute table has six attributes: id_device, date, by_min (minute), total (total value in a minute), count (number of readings in a minute) and context (the last reading place). The by_hour table is similar to the by_minute table. Finally, the by_day table has four attributes: id_device, date, total (total value in a day) and count (number of readings in a day).

The context extraction module captures contextual metadata. Context is important because it allows using the environmental information in order to facilitate decision making when necessary. Context is considered as a dynamic process, constructed through the interaction of devices/sensors around the world, and over time. Several applications can use contextual information, such as location, time and data source, to make recommendations. In this scenario, the context extraction module executes three parallel processes, similar to the summarization module, to get for instance the location of devices in the last minute, the last hour and the last day.

## 5. Implementation of the DDS

In this section, the implementation of the proposed DDS for IoT middleware is presented with the discussion of component choices. The architecture implementation acts as a bridge between the proposed architecture and a set of implementation technologies, providing key elements in conformance to the prescriptions and constraints of the architecture. In this context, the DDS implementation must define which technology is used to support the architecture features such as parallel and distributed processing. The implemented prototype is an important tool for validating the specification of requirements, the design of the DDS and the expected performance.

### 5.1. Used Technologies

To implement the different components and modules of the DDS architecture, we had to integrate some technologies. For the data collection component, the Apache Kafka platform was used, since this platform satisfies the requirements of the data collection component regarding the implementation of the capture module. The filtering and metadata modules are implemented with the Apache Storm framework, which is projected to process data in real time. To store data produced by the data collection component, the Apache Cassandra database is also a choice that also allows the time series compaction module to be implemented by this database. Apache Cassandra supports the implementation of different modules of the data aggregation component. Since our proposal requires the definition of a distributed architecture capable of processing different tasks in parallel, the chosen technologies to be used by the DDS prototype must support distributed and parallel processing.

Apache Kafka is an open source, distributed, publish-subscribed, messaging system, often used as a kernel for architectures that deal with data streams [41]. Designed with features such as persistent messaging, high performance, easy distribution, the ability to support multiple customers and perform real-time processing, Kafka has three main elements [42]: topics, producers and consumers. A topic is simply an abstract place where messages are published, while Kafka producers are processes that publish or create messages for a Kafka topic. Consumers access a topic and process the messages posted therein.

Apache Storm is an open source, distributed, real-time computation system [43]. A Storm cluster is composed by a Nimbus server, one Supervisor and Zookeeper. The Nimbus is responsible for distributing processes across the cluster and monitoring it. The Storm cluster may have one or more supervisor nodes, where processes are executed. The Supervisor nodes communicate with the Nimbus through the Zookeeper [44]. The Nimbus sends signals to the supervisor to start or stop processes. The Zookeeper is a distributed high performance coordination service to maintain information regarding configuration, delegation, to providing distributed synchronization and service groups.

Apache Cassandra is a NoSQL database system that intends to achieve the following requirements: high availability, high performance, reliability and a scalable architecture to support large and ever-increasing amounts of data [45,46]. Although Cassandra follows a column-oriented model, thus representing a columnar database, it internally works based on the key-value concept. Cassandra is the choice for data storage in our proposal because it includes a strategy for handling time series called ‘DateTieredCompactionStrategy’, which can improve the performance of the time series computations.

Finally, the Streamparse platform was also considered in our prototype implementation because it lets users run Python code modules to deal with real-time streams of data via the Apache Storm [47]. This tool takes advantage of parallel and distributed processing provided by the Apache Storm, and their combination is an important alternative for real-time processing systems since Streamparse offers the map/reduce processing style in real time for data streams. Moreover, it can be a way to scale Python processes with high parallelism. In our proposal prototype, this tool allows the integration of Kafka, Storm and Python libraries; tools that comprise the core of the current data processing infrastructure, able to process a huge amount of time series data.

### 5.2. Implementation of Data Collection and Data Aggregation Components

**Figure 7:**
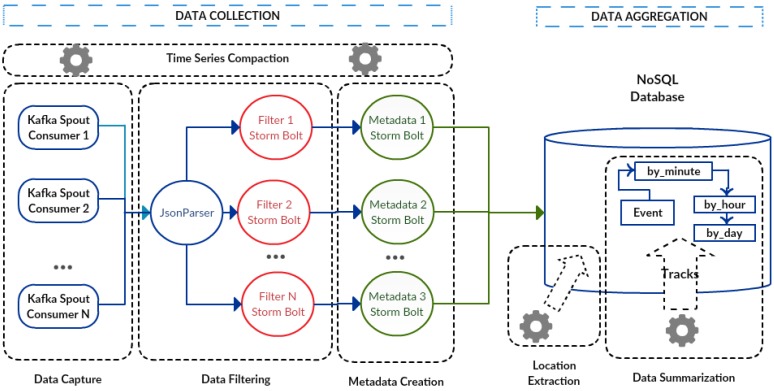
DDS topology.

Figure 7 presents the implementation architecture with each data collection and aggregation components. The first one is the data capture module, in which every captured message is stored into a topic, a kind of database. These messages are stored without any transformation and can be used as historical data to be reprocessed for futures analyses. More than one job instance for reprocessing can be executed, but each one starting from a different position in the historical dataset. Additionally, this feature increases the parallelism level and allows replaying data history very quickly.

The data capture module comprises an arbitrary number of consumer processes implemented with the Kafka message system, called Kafkaspout [42]. Each module is distributed and executed in parallel using the different nodes of the cluster, which allows the processes that make up each module to be executed simultaneously.

The data filtering module has two well-defined stages in its topology: JsonParser and filters. These processes are implemented using the Apache Storm real-time processing system together with Kafka. The JsonParser bolts [43] are processes that receive messages from the consumer KafkaSpout and divide these JSON messages into smaller units. The filter bolts implement the filtering of the messages, guaranteeing the integrity of the domain, by verifying for instance whether the values of sensor readings are within the allowable range. After the message filtering, they are stored in a Cassandra database [48].

The metadata creation module was implemented with the Apache Storm/Kafka architecture [49], where the metadata bolts allow the creation of metadata from messages already filtered. The metadata process is the third stage in the Storm/Kafka topology. It is noticeable that the three stages of the topology (JsonParser processes, filters and metadata) can be executed in parallel. To deal with the variety of collected data from IoT environments, two tables are defined to store the unit of measurements. The first table has the source unit, the International System of Units (SI) equivalent and the conversion formula to transform from the original to the SI unit. The second table comprises the SI unit, the source unit and the conversion formula to transform from the SI to the original unit. In this context, the metadata process uses the first table to convert the source measurement value to the SI unit and the second table for the inverse conversion. For example, it is possible to convert from Celsius degrees to Kelvin degrees, using the conversion formulas for each case.

Finally, to record the data in the Cassandra database, a key was developed that is composed of the timestamp of the sensor/device reading and the sensor/device identifier. This key guarantees the singularity of the data, once the sensor/device readings, together with the sensor/device identifier, are distinct. In addition, to increase the rate of the insertion of the data in the proposed service, a micro-batching insertion approach [50] is used, which accumulates the reception of messages over a short interval of time (2 s, for example). Later, these messages can be inserted together in the database, which improves the corresponding insertion rate. The data are recorded in the event table with the following attributes: timestamp, id_device, status, id_variable, value and localization.

#### 5.2.1. Configuration of the Cassandra Database

The Cassandra database was configured to support time series data coming from the IoT middleware. First, the data are stored in a regular column family, but with a specialized strategy for compaction called “DateTieredCompactionStrategy”, which is used by the time series compaction module. This compaction strategy is used in every column family that stores time series data in the data collection and data aggregation components.

For each IoT middleware integrated with the proposed DDS, a key space is created in Cassandra. This is important since it is a measure that provides better organization, both by allowing the separation of data into different key spaces, although some data obey the same schema, and by setting a replication factor to ensure reliability and fault tolerance. In our validation, we adopt the value of three for this factor.

#### 5.2.2. Kafka Consumer Configuration

**Figure 8:**
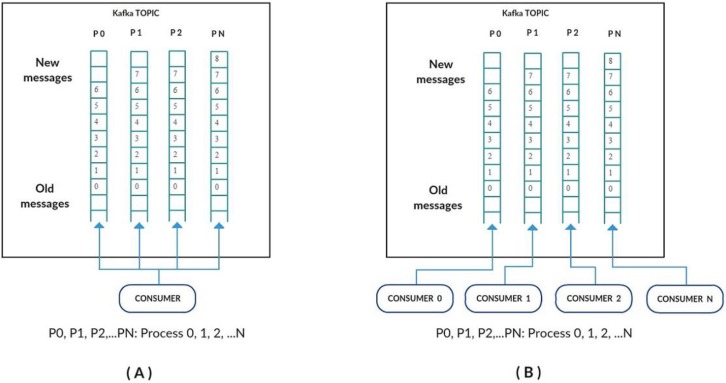
Kafka Consumer Configuration. In (**A**), a Kafka consumer reads all data partitions (P0, P1, P2, ... PN) as data arrive and this sole consumer limits the overall capacity even considering the existence of parallel partitions to manage the volume of data coming from a large network of devices under the control of an IoT middleware, while in (**B**), multiple Kafka consumers operate each one dedicated to reading its specific data partition only, thus leveraging the parallelism in partition reading.

To process the large volume of data collected into a topic, the Kafka consumers have to be properly configured. In general, a Kafka consumer reads data partitions (P0, P1, P2, ... PN) as the data arrive, as shown in Figure 8A. However, even achieving the reading of parallel partitions, the degree of Kafka parallelism is not enough to manage a large volume of data provided on a large network of devices/sensors under the control of an IoT middleware.

In order to improve parallelism, a modification was introduced in how the partitions are read by the Kafka consumer, by defining a consumer for each existing partition, as shown in Figure 8B. This modification increases the degree of parallelism in partition reading, since instead of having only one consumer reading multiple partitions, now there is one consumer dedicated to reading only one partition.

### 5.3. Implementation Details of Data Aggregation Components

The data aggregation component was implemented with two modules (Figure 7): data summarization and context extraction.

The data summarization module implements the summarization of data, which are stored in the event table by the data collection component. The summarization process establishes connections with the Cassandra database and performs queries over the event table. This program implements three processes respectively for summarizing data either by minute, by hour or by day intervals. Results from these processes are stored in summarized tables that contain the number of messages received and the total value of the measurements during a period of time. This allows different calculations from the summarized data, such as the average for a period of time.

The context extraction module implements the location extraction, which is especially useful when a device/sensor is attached to a moving thing such as a car or a bike. The device’s/sensor’s location either is frequently changing, or its location can be relatively static according to the object mobility. In addition, data coming from remote sensors, such as images and video from cameras and satellites, require the location from where data are coming [51]. Thus, the extraction module is implemented similarly to the summarization module, using the event table as a data source. In this case, the process aims at extracting the location attribute from the event table to store into the summarized tables. After the location field is selected, the process fetches the last localization of device/sensor, i.e., when a device/sensor reading was done.

## 6. Case Study: Smart Home System Simulation

To evaluate the performance of the designed DDS, this section presents measurements from two experiments comprising the simulation of a home automation IoT instance. The first simulation processes data generated by thousand of homes with home appliances and devices managed by the UIoT middleware [52]. In this experiment, we aim to evaluate the two DDS components (data collection and data aggregation). Hereinafter, this experiment is named the UIoT-DDS study. The second simulation is set up to compare the DDS data collector to a similar collector in the Kaa middleware [11], in a home automation IoT similar to the first simulation. The objective of this second study is to show the performance of the DDS data collector compared to a Kaa collector in terms of capacity to ingest a huge volume of data. Hereinafter, this second experiment is named the Kaa collector study. In both experiments the emulated smart home system includes seven devices that are listed in Table 1.

### 6.1. UIoT-DDS Study

To achieve the case study tests for data collection and data aggregation, we implemented a computational environment with a cluster composed of four machines, each with the following specifications: Intel Xeon 2.5-GHz processor with eight cores, 8 GB of RAM, 500 GB of HD and 1 Gb Ethernet. The operating system Ubuntu Linux 14.04 was configured in each nodes with Apache Kafka 0.8.2, Apache Storm 0.9.5, Apache Cassandra 2.2.6 and Streamparse 2.0.0.

For message interchange simulation between the UIoT and the DDS, Kafka producers are programed to create eight million messages simultaneously, each message having 160 bytes. Table 2 shows the number of homes and the number of devices that the simulation represents. Moreover, it presents the data volume for different periods. The calculation is based on an operation that proceeds by reading devices every 10 s and sending the corresponding 160 byte messages.

In order to support the huge data volume, the distributed data service was projected to function on a computational cluster based on a messaging system. Then, the DDS is cluster based and uses publish-subscribe messaging to handle read and write operations. One of the cluster nodes is selected as a master server node for the data collector. The other cluster nodes are slave servers that receive messages from the master server for storing and processing purposes.

**Figure 9:**
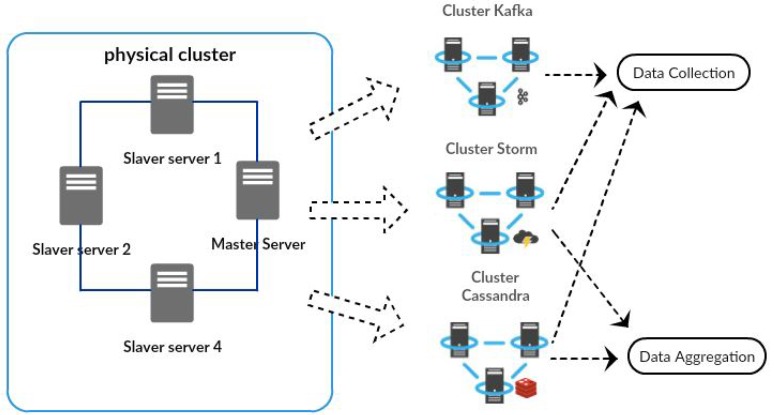
Computational environment of DDS.

It is important to note that a physical cluster, as a shown in Figure 9, was implemented. This cluster internally supports three virtual clusters, respectively: the Kafka, the Storm and the Cassandra cluster.

The Kafka cluster was set up on four machines, and this cluster was tested with a replication factor of four. The replication factor is proportional to the number of nodes in the Kafka cluster. In order to get the maximum parallel processing, the replication factor must be equal to the number of nodes in the Kafka cluster. In order to scale the Kafka cluster horizontally, it was necessary to add more partitions and spread data onto more machines.

The Storm cluster was implemented with four machines; one of them reserved to operate as the master node (Nimbus) and the other three as slave nodes (Supervisor). In the Storm architecture, the Nimbus server is the master server for the Storm cluster, responsible for distributing and managing the processing load. In this scenario, the supervisors are slave servers, which have the task of processing data according to their processing power.

The Cassandra cluster was configured in a multi-node cluster with four nodes. Instead of following a master/slave architecture, the Cassandra cluster is a storage system organized as a peer-to-peer network. Thus, one of the nodes was selected as a seed node, and this node is marked as a stared node, which knows at least one node of the Cassandra ring.

In our prototypical implementation, the Kafka, Storm and Cassandra clusters are used by the DDS data collection component while the Storm and Cassandra clusters are used by the DDS data aggregation component.

### 6.2. Kaa Collector Study

To conduct the case study tests, we implemented a computational environment with a cluster composed of four virtual machines, each with the following specifications: Intel Xeon 2.5 GHz processor with four cores, 8 GB of RAM, 50 GB of HD and 1 Gb Ethernet. The operating system Ubuntu Linux 14.04 was configured in each node with Kaa v0.10.0. Besides this, the Kaa cluster internally is supported by a distributed MongoDB as its NoSQL database and the Kaa framework set in each cluster node.

**Figure 10:**
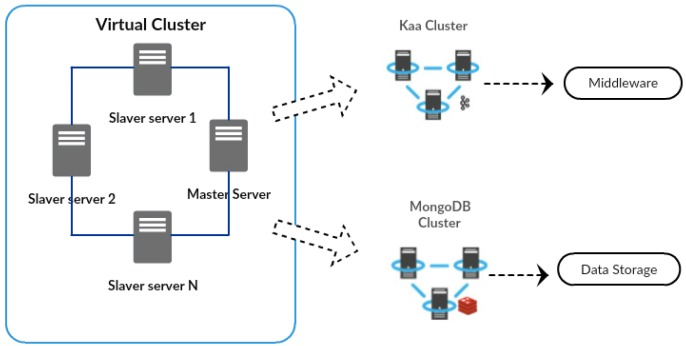
Computational environment of DDS.

It is worth highlighting that the cluster environment was implemented over four virtual machines, as shown in Figure 10. This cluster internally supports two virtual clusters, respectively the Kaa and the MongoDB cluster.

In this scenario, the simulation concerns a thousand endpoints sending millions of messages (logs) simultaneously at a constant rate. Because of the synchronous nature of Kaa middleware, the message generation is only done synchronously. The endpoint log structure includes data regarding the ID of the source, service description, transmission timestamps and measurement value. The format of the log messages is customized and preconfigured in a log schema, and the message size is 50 bytes. When the Kaa server receives a log message, it stores the log in the MongoDB database and then replies with an ack to the endpoint.

Similarly to the UIoT-DDS study, the Kaa collector study is implemented with Kafka producers creating eight million messages simultaneously. Table 3 shows the number of homes and the number of devices that the simulation represents. Moreover, it presents the data volume for different periods. The calculation is based on an operation that proceeds by reading devices every 10 s and sending the corresponding 50-byte messages.

## 7. Results

The results are divided according to the two described studies. In the case of the UIoT-DDS simulation results, data collection and data aggregation were evaluated, while in the Kaa simulation, only data collection was evaluated. In order to ensure equity, the DDS and Kaa environment implementations were carried out in a similar fashion, in terms of the quantity of cluster nodes, memory, processing power and operational systems. However, there were some differences. For example, the DDS environment was implemented by physical computers, while the Kaa environment was implemented by virtual machines.

### 7.1. DDS Results

**Figure 11:**
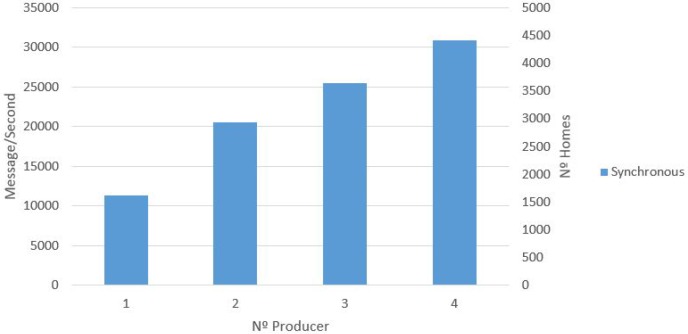
UIoT data producer: synchronous.

**Figure 12:**
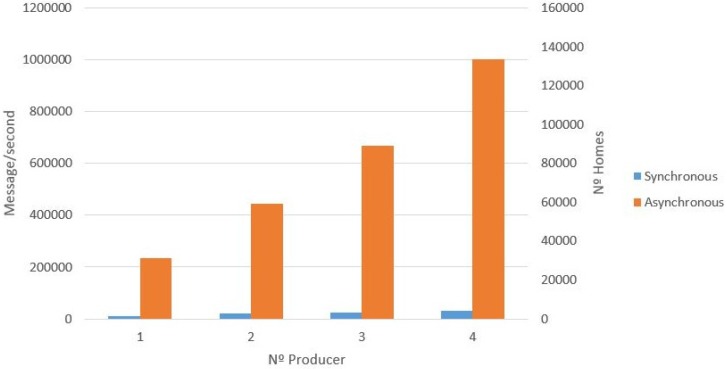
UIoT data producer (synchronous and asynchronous).

The DDS simulation involved the creation of both synchronous and asynchronous messages. The results of this simulation are presented, with the number of messages created per second by a different number of producers which are either broker or server nodes. This process was tested in an incremental way, from one producer to four producers. Figure 11 presents the results, with the synchronous producer showing that the performance is proportional to the number of producers being executed. In addition, the number of homes that can be supported in the synchronous scenario is presented. For example, for two producers, the number of messages per second is 20,000 (2934 homes). Figure 12 presents the results of the asynchronous producer compared to the synchronous producer. Similar to the synchronous producer experiment, this process was tested in an incremental way, from one producer (broker or server node) to four producers.

After the data aggregation processes completed the preprocessing, data coming from the event table were stored in the summarized tables. To show the performance improvement in the query response time using the aggregation approach, we refer to the query time using the event table where every message is stored and collected by the data collection, and we compare this result with the query time of the aggregation processes from the summarized table. The Event table stores every filtered message coming from an IoT middleware with any type of aggregation. Table 4 shows that querying data from the event table is more expensive compared to querying from the summarized table. This becomes more evident over a longer period.

These results show that our DDS prototype in Kafka performs very well with multiple brokers in the cluster. In the case study presented, Kafka supports the ingestion of millions of IoT messages created by the Kafka producer, thus supporting high stress requests. Meanwhile, the KafkaSpout consumer easily supports the ingestion of data from the producers. This is a result of the multiple partitions created in a Kafka topic. The partitions enable read and write operations in parallel. In general, while some partitions are read by the Kafka consumer, other partitions are written by the Kafka producer. Furthermore, the results show that Storm adequately performs the defined DDS topology, enabling the mounting of a cluster to process data in a distributed and parallel way, using the processing resources of every cluster node to process all of the data in real time.

### 7.2. Kaa Results

**Figure 13:**
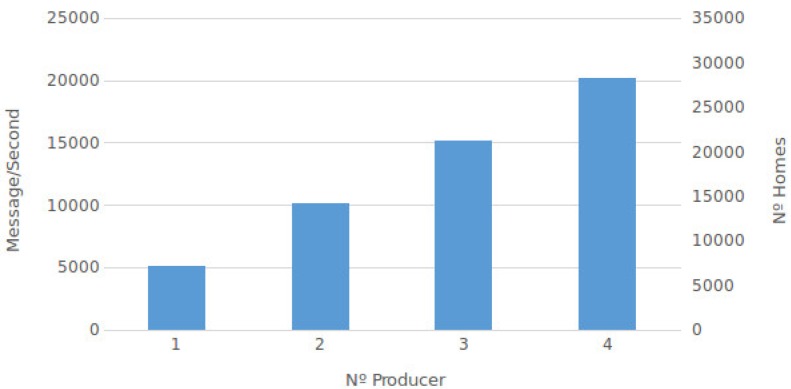
Kaa collector results.

Unlike the UIoT-DDS simulation, the Kaa simulation involved exclusively the creation of synchronous messages. This is due to the fact that data collection in a Kaa platform can only store the received messages in the database and then reply with a confirmation or acknowledgment back to the endpoint. Figure 13 displays the relationship between the message receiving speed and the number of homes supported by different numbers of Kaa nodes.

The results of this Kaa simulation are presented with the number of messages created per second by a different number of producers. This process was tested in an incremental way, from one producer to four producers. While the producer is incremented, the number of Kaa nodes is also incremented proportionally. Figure 13 presents the results showing that performance is proportional to the number of producers being executed. In addition, the number of homes that can be supported is shown. For example, for two producers, the number of messages per second is 10,094 (1442 homes).

## 8. Discussion

In this section, we intend to analyze first the data collection and data aggregation results of the UIoT-DDS study, second, the Kaa collector results and, finally, the data collection comparing the UIoT-DDS to the Kaa collector in terms of data ingestion of a huge data volume. This comparison shows the better performance of DDS when facing a huge volume of data coming from different sources. It is important to highlight that for the sake of fairness, this comparison is done in terms of data ingesting when UIoT-DDS operates synchronously, since the Kaa collector only operates in synchronous mode.

In the first case study (UIoT-DDS), we can see the performance of the asynchronous producer presenting better results than the synchronous one (see Figure 12). The rapid data ingestion by the asynchronous producer is due to the fact that there is no need to confirm that a message was successfully received. This allows sending messages without having to wait for an answer regarding a previous message. For this reason, the data ingestion for the asynchronous simulation is faster than a synchronous equivalent. The results show that the data collection can support data ingestion at the rate of a million messages per second. For example, for two producers, the number of messages per second is 400,000 (63,492 homes). Figure 12 shows the scalability regarding data ingestion by the proposed setup, meaning that the data ingestion by the DDS can support a huge number of devices sending data at the same time, since it is possible to add more brokers to the cluster topology.

About the data aggregation process, the event and summarized tables do not have any particular configuration, such as index creation. Table 4 presents an event column, which represents the query time over a period of time. In this context, since one million devices/sensors can generate about 8640 million records in one day, a query over this volume of data can take a long time to be processed. With this in mind, querying data by day is slower compared to querying by hour or by minute. It is clearly observed that the query time over summarized data is very fast, and apparently, it has a constant processing time. This is a consequence of a decreased volume of data and the respective preprocessing. This means that there are fewer rows to consider, and thus, less processing is required to get a response.

In the second case study (Kaa collector), we can see the performance of data collection using the Kaa platform whose scalability is illustrated in Figure 13. Incrementing the number of Kaa nodes, the data collection capacity is incremented proportionally. In this context, the average number of collected messages is about five thousand messages per second, considering the increment of the number of producers.

Comparing data collection results between the DDS and Kaa collectors, we can see that DDS collection shows better results compared with data collection within the Kaa platform. For example, for just one node, the DDS can collect 11 thousand messages per second, and the Kaa collector can collect about five thousand messages per second. Another example is when comparing with three nodes, the result is about 25 and 15 thousand messages per second, respectively. It is important to again highlight that this comparison is done with DDS synchronous producers. Comparing the DDS asynchronous producer with the Kaa collector is not fair, given the evident better performance of the DDS collection data due to the asynchronous operation mode. In this case, for example, for four nodes, the DDS can collect about one million messages per second, while the Kaa collector can collect just about 20 thousand messages per second.

## 9. Conclusions and Future Works

The next generation of the Internet is moving in an important developmental direction, as the IoT increasingly captures the attention of industry and academia. Selecting and applying database middleware technology, in a reasonable way, is a key question to solving the problem of IoT data management for a massive volume of data generated in real time.

In this context, our results show that the designed DDS supports data management for IoT middleware performs well in collecting and retrieving data from an IoT middleware. The choice of components and their articulation for collaborative data treatment are key factors for high velocity data collecting and analyzing. Besides this, the proposed DDS is able to process a variety of data coming from different IoT environments through a specific communications interface and metadata creation modules. Finally, the proposed DDS was shown to have better performance compared with a typical data collector, the Kaa middleware.

In our proposed DDS prototype, the Kafka database has shown adequate performance in collecting massive amounts of messages managed by the UIoT middleware in the simulated setup. Repetitive tests show that the performance of Kafka is optimal regarding the utilization of a Storm cluster for real-time processing. In this configuration, the data aggregation component was shown to be efficient in querying incoming data from millions of IoT devices, which are expected in large IoT networks. The results show that the proposed loosely coupled DDS supports a high stress load of incoming data from an IoT middleware managing millions of IoT devices to collect and process the data they create. Loosely coupling these systems seems to be a reasonable approach if we consider that there are data management tasks common to various IoT middleware systems; these tasks being factored and separated from the IoT control middleware can be offered as a distributed software service that can be independently developed and, if needed, can be transformed to a software library for tightly-coupled systems. Loose coupling can also be important in providing interoperability among different IoT middleware and IoT applications.

Future work may include further implementation and a more detailed performance evaluation of the data collection and the data aggregation components. In addition, massive data analysis allowing learning from the IoT environment, as well as data prediction based on sensor serial time data are also to be studied. Finally, we intend to implement a real-life, smart environment, to collect and process actual data produced by devices and sensors in this environment.

## Figures and Tables

**Table 1:** Devices of the home automation system.

Device Name	State Variable (SV)	SV Allowed Values	Reading Circuit	Write Circuit
Television	Channel	1 to 1000	-	IR circuit
	Volume	1 to 100	-	IR circuit
	Power	On, Off	-	IR circuit
Curtain	Movement	Open, Close	-	IR circuit
Microwave oven	Power	On, Off	Voltage sensor	Switch circuit
Coffee pot	Power	On, Off	Voltage sensor	Switch circuit
Gate	Movement	Open, Close, Stop	-	IR circuit
	Distance	2 to 700	Distance detector	-
Alarm clock	Power	On, Off	Voltage sensor	Switch circuit
Stereo	Power	On, Off	-	Switch circuit
	Volume	1 to 100	-	IR circuit
	Station	1 to 6	-	IR circuit

**Table 2:** Number of devices for UIoT-DDS.

Number of Producers	Number of Homes	Day	Month	Year
1	33,600	0.29 TB	8.87 TB	107.92 TB
2	63,492	0.56 TB	16.76 TB	203.96 TB
3	95,238	0.84 TB	25.15 TB	305.92 TB
4	142,857	1.26 TB	37.72 TB	458.91 TB

**Table 3:** Number of devices for Kaa.

No. of Prod.	No. of Homes	Day	Month	Year
1	33,600	0.09 TB	2.86 TB	33.73 TB
2	63,492	0.17 TB	5.41 TB	63.74 TB
3	95,238	0.26 TB	8.12 TB	95.61 TB
4	142,857	0.39 TB	12.18 TB	143.41 TB

**Table 4:** Query time over the event and the summarized tables.

	Query Time	
**Period**	**Event (s)**	**Summarized (ms)**
By minute	1.51	4
By hour	2.12	3
By day	43.94	3

## Abbreviations

The following abbreviations are used in this manuscript:

IoT: Internet of Things
DDS: Distributed Data Service
NoSQL: Not only SQL
WSO2: Web Services oxygenated
CUPUS: CloUd-based Publish-Subscribe
REST: Representational State Transfer
ITS: Intelligent Transportation System
IC: Integrated Circuits
SV: State Variable
IR: Infrared
RFID: Radio Frequency Identification
GPS: Global Positioning System
UDP: User Datagram Protocol
X-GSN: Extended Global Sensor Networks
JSON: JavaScript Object Notation
XML: EXtensible Markup Language
HTTP: Hypertext Transfer Protocol
UIoT: Universal Internet of Things
